# A systematic review of optical coherence tomography findings in adults with mild traumatic brain injury

**DOI:** 10.1038/s41433-023-02845-w

**Published:** 2024-01-18

**Authors:** Hannah S. Lyons, Matilde Sassani, Yousef Hyder, James L. Mitchell, Mark Thaller, Susan P. Mollan, Alexandra J. Sinclair, mTBI Predict Consortium, Alexandra Sinclair, Aliza Finch, Adam Hampshire, Alice Sitch, Ali Mazaheri, Andrew Bagshaw, Andy Palmer, Asha Strom, Alice Waitt, Andreas Yiangou, Ahmed Abdel-Hay, Alexander Bennett, Amy Clark, Angus Hunter, Barry Seemungal, Caroline Witton, Caroline Dooley, Deborah Bird, Davinia Fernandez-Espejo, Dave Smith, Dan Ford, Daniel Sherwood, Donna Holding, Duncan Wilson, Edward Palmer, John Golding, Hamid Dehghani, Hyojin Park, Hannah Lyons, Hazel Smith, Helen Brunger, Henrietta Ellis, Iman Idrees, Ian Varley, Jessica Hubbard, Jun Cao, Jon Deeks, James Mitchell, Jan Novak, Jamie Pringle, John Terry, Jack Rogers, Tim Read, Jessikah Fildes, Karen Mullinger, Lisa Hill, Marco Aurisicchio, Mark Thaller, Martin Wilson, Mark Pearce, Matilde Sassani, Matthew Brookes, Mohammad Mahmud, Ray Rayhan, Ned Jenkinson, Niki Karavitaki, Nick Capewell, Olivia Grech, Ole Jensen, Pete Hellyer, Philip Woodgate, Sebastian Coleman, Raymond Reynolds, Richard J. Blanch, Katie Morris, Ryan Ottridge, Rachel Upthegrove, Ronan Dardis, Ruwan Wanni Arachchige, Sarah Berhane, Sam Lucas, Sophie Prosser, Shayan Sharifi, Shreshth Dharm-Datta, Susan Mollan, Toby Ellmers, Tara Ghafari, Tony Goldstone, Waheeda Hawa, Yidian Gao, Richard J. Blanch

**Affiliations:** 1 Translational Brain Science, Institute of Metabolism and Systems Research, College of Medical and Dental Sciences, University of Birmingham, Birmingham, B15 2TT UK; 2 Department of Neurology, University Hospitals Birmingham NHS Foundation Trust, Queen Elizabeth Hospital, Birmingham, B15 2WB UK; 3 Academic Department of Medical Rehabilitation, Defence Medical Rehabilitation, Stanford Hall, Loughborough, UK; 4 Department of Ophthalmology, University Hospitals Birmingham NHS Foundation Trust, West Midlands, UK; 5 Royal Centre for Defence Medicine, Birmingham, UK; 6 Neuroscience and Ophthalmology Research Group, Institute of Inflammation and Ageing, University of Birmingham, Birmingham, UK; 7 University of Birmingham, Birmingham, UK; 8 Imperial College, London, UK; 9 Aston University, Birmingham, UK; 10 University Hospitals Birmingham, Birmingham, UK; 11 Defence Medical Rehabilition Centre, Loughborough, UK; 12 Nottingham Trent University, Nottingham, UK; 13 Nottingham University, Nottingham, UK; 14 Defence Medical Services, Lichfield, UK; 15 University of Westminster, London, UK; 16 University of Nottingham, Nottingham, UK; 17 King’s College London, London, UK; 18 University Hospitals Coventry & Warwickshire, Coventry, UK

**Keywords:** Predictive markers, Retina, Trauma, Brain injuries

## Abstract

Mild traumatic brain injury (mTBI) is common with many patients suffering disabling long-term sequelae, with visual symptoms frequently reported. There are no objective biomarkers of mTBI that are routinely used in clinical practice. Optical coherence tomography (OCT) has been used in mTBI research, as it enables visualisation of the neuroretina, allowing measurement of the retinal nerve fibre layer and ganglion cell layer. This systematic review aims to appraise the available literature and assess whether there are significant changes within the retinal nerve fibre layer and ganglion cell layer in subjects after mTBI. A systematic review was carried out in accordance with PRISMA guidelines and registered with PROSPERO (Number: CRD42022360498). Four databases were searched for relevant literature published from inception until 1 September 2022. Abstracts and full texts were screened by three independent reviewers. Initial screening of databases yielded 341 publications, of these, three fulfilled all the criteria for inclusion. All three studies showed thinning of the retinal nerve fibre layer, whereas there were no significant changes in the ganglion cell layer. This systematic review demonstrated that thinning of the retinal nerve fibre layer (but not of the ganglion cell layer) is associated with mTBI. It provides preliminary evidence for the use of the retinal nerve fibre layer as a potential biomarker of damage to the visual system in mTBI. Further prospective longitudinal studies ensuring uniform diagnosis and accurate phenotyping of mTBI are needed to understand the effects on the visual system and potential of OCT as a prognostic biomarker.

## Abstract (zh)

轻度颅脑损伤(mTBI)常见于许多患有长期残疾后遗症的患者, 常有视觉症状报道。在临床实践中没有常规使用的描述mTBI的客观生物标志物。相干光断层扫描(OCT)已用于mTBI研究, 因为它可以实现神经视网膜可视化, 可以定量观察视网膜神经纤维层和神经节细胞层的变化。本综述旨在评估现有文献, 并评估mTBI受试者的视网膜神经纤维层和神经节细胞层是否有显著变化。

本系统综述按照PRISMA指南开展, 并在PROSPERO注册(编号: CRD42022360498)。在四个数据库中检索从开始到2022年9月1日发表的相关文献。摘要和全文由三位独立评审人进行筛选。

对数据库初步筛选产出了341个文献, 其中三份符合所有纳入标准。这三项研究均显示视网膜神经纤维层变薄, 而神经节细胞层没有明显变化。

本综述表明, 视网膜神经纤维层变薄(而不是神经节细胞层)与mTBI有关。它为视网膜神经纤维层作为mTBI视觉系统损伤的潜在生物标志物提供了初步证据。需要进一步的前瞻性纵向研究来确保mTBI的统一诊断和准确表型, 以了解OCT作为预后生物标志物对视觉系统的影响和潜力。

## Background

Traumatic brain injury (TBI) is a major public health problem worldwide [1]. It is defined as an alteration in brain function, or any other evidence of brain pathology, caused by an external force [1, 2]. It can be categorised by severity (mild, moderate and severe), penetrating or non-penetrating (open or closed), and blast or non-blast. The main classifications based on severity are: Veterans Affairs Department of Defense (VA/DoD) [3], World Health Organisation (WHO) [4] and Mayo [5].

Mild traumatic brain injury (mTBI) is common with nearly 1.2 million Emergency Department visits annually, and encompasses approximately 85% of those admitted with a head injury in England and Wales [6]. Although classified as ‘mild’, many of these individuals suffer significant disabling long-term sequalae, including posttraumatic headache, vestibular, visual and cognitive dysfunction, as well as mental health problems [4]. The National Institutes of Health declared mTBI a major public health problem because of the frequency of underdiagnosis and the high societal burden [7]. Most TBI in Europe are caused by road traffic accidents and falls; 63% of patients are adults between 16 and 64 years old [8, 9]. Nonetheless, TBI is also an important cause of morbidity in the elderly population, who are more prone to recurrent falls, with 80–84 year olds having one of the highest incidence rates of head injury, second to 15–19 year olds [9, 10]. A large proportion of TBI affects working-age adults, with 50% of patients complaining of functional limitations 12 months after the event. It is also an indirect cause of unemployment, productivity loss and work limitations [11, 12]. In the United States, the estimated economic impact of TBI in 2006 was $9.2 billion direct costs *per annum* and $51.2 billion indirect costs from missed work and lost productivity [13].

In TBI, shear forces cause a primary insult to neurons, axons, glia and blood vessels; this initial damage activates a wave of metabolic and inflammatory cascades causing secondary injury [14]. In addition to the immediate effects, several studies suggest an association between a history of TBI and increased risk of Alzheimer disease [15, 16], Parkinson disease [17], amyotrophic lateral sclerosis [18], cognitive impairment [19], and multiple sclerosis [20], suggesting progressive neurodegeneration over time after the primary and secondary injuries [21]. TBI may therefore be better classified as a disease process, not an isolated event.

The impact on the visual system may be one of the most disabling manifestations of mTBI [22]. As more than 30 brain areas and cranial nerves two to eight affect visual functioning, it is unsurprising that mTBI causes visual dysfunction [14]. The retinal nerve fibre layer (RNFL) may also be injured by the primary and secondary brain insults [14, 23].

Visual dysfunction can be difficult to identify after mTBI because the development of visual sequalae or patient reporting may be delayed. Visual changes include visual field defects [24], vergence dysfunction [25], reading dysfunction [26], abnormal pupillary responses [27] and accommodative dysfunction [28]. In addition, after mTBI, 20–40% of patients suffer deficits in smooth pursuit, tracking, and pursuit initiation [29].

At present there are no objective measures that predict the likelihood of developing visual complications after mTBI. One potential measure is optical coherence tomography (OCT) imaging, a non-invasive and rapid modality that can perform serial measurements of the RNFL and macula ganglion cell layer (GCL) rendering it ideal for prolonged longitudinal studies [30, 31]. Whilst retinal thickness changes after mTBI are not well understood, OCT may allow detection of early structural changes, as well as revealing subsequent damage to neuronal tissue.

In this review we aim to appraise systematically all available literature reporting OCT RNFL and GCL measurements after mTBI to assess the potential of OCT as a biomarker for visual damage.

## Methods

### Databases and search strategy

This systematic review was registered on PROSPERO (Number: CRD42022360498) and was conducted according to PRISMA guidelines [32]. Four databases (PubMed, EMBASE, clinicaltrials.gov and Cochrane Library) were searched for relevant literature published from inception until 1 September 2022. Search terms consisted of different variations of keyworks, including (1) Trauma* brain injury OR TBI OR head injury OR brain injury OR concuss* OR “traumatic optic neuropathy”; AND (2) “Optical coherence tomography” OR OCT; AND (3) “Retinal nerve fibre layer” OR RNFL OR GCL OR GCIPL OR “ganglion cell”. The search strategy was identical in all databases, with minor adaptation to the coding to suit individual database settings. All citations were imported and managed using EndNote 20.

### Inclusion and exclusion criteria

The inclusion and exclusion criteria are shown in Table 1. Review articles were excluded; however, their references were also screened to search for any additional publication that might have fitted inclusion criteria.

**Table 1:** Inclusion and exclusion criteria.

Inclusion criteria	Exclusion criteria
Age of study population 18 years and older	Papers not available in English
Male and female sex	Review articles and case reports with 1 or 2 patients
Human study	Duplicate papers
Segmented OCT with GCL and/or RNFL thicknesses	Time-domain OCT
Mild TBI based on DoD, WHO or Mayo criteria.	Brain disease acquired in a manner unrelated to trauma
	Animal, cellular or in vitro studies
	Mode of injury described as “concussion” but not consistent with formal definitions of mTBI)
	Moderate, severe or unclassified TBI
	Conference proceedings.

*OCT* optical coherence tomography, *GCL* ganglion cell layer, *RNFL* retinal nerve fibre layer, *mTBI* mild traumatic brain injury, *DoD* department of defense, *WHO* world health organisation.

### Papers selection and data extraction

Three authors (HL, MS, YH) worked independently and each screened all titles and abstracts for relevant articles; arbitration of disagreements was performed by an independent senior fourth author (RB). Two authors (HL and MS) then worked independently to review full-text articles of all articles marked relevant to confirm eligibility based on abovementioned criteria; arbitration of disagreements was performed by an independent senior fourth author (RB). Two authors (HL and MS) collected the data for the included studies and assessed risk of bias independently.

Methodological risk of bias for each study was appraised using the Newcastle-Ottawa Scale, which was designed to assess risk of bias for case-control and cohort studies on the basis of three main domains (selection, comparability and outcome) and eight sub-domains [33]. The following data were extracted and reported: (1) Study design; (2) Geographical location of the study; (3) Study setting (single centre or multicentre); (4) Sample size; (5) Cohort age expressed as mean and standard deviation (SD); (6) Percentage of female participants; (7) Military or civilian cohort; (8) Severity of TBI reported; (9) Diagnostic criteria applied; (10) Mode of injury; (11) Time since injury; (12) Type of OCT utilised; (13) Number of time points; (14) Presence of control group, (15) Patient-reported outcome measures; 16) Resulting statistics for reported RNFL and GCL changes.

## Results

### Search results

**Fig. 1:**
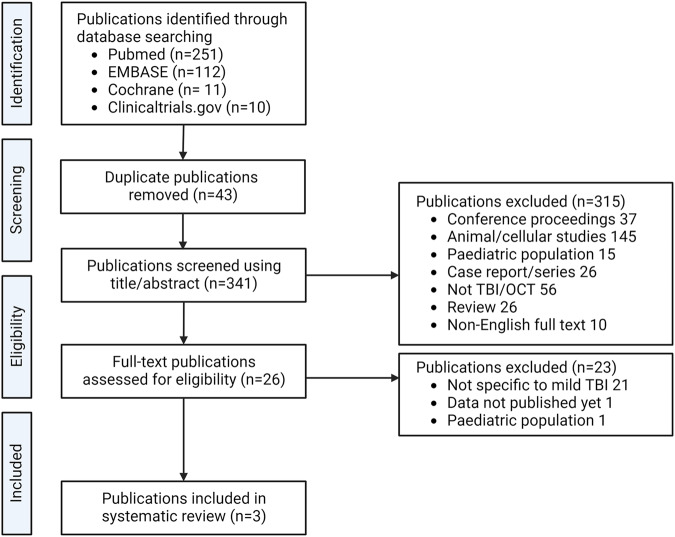
Consort diagram outlining searches, screening and study selection for inclusion.

After removal of duplicates (43), database searches yielded 341 publications. Of these, 315 were excluded based on their titles and abstracts: 63 were not primary research articles (i.e. conference proceedings or reviews), 145 were animal studies, 15 assessed a paediatric cohort, 26 were case reports with fewer than three patients, 56 assessed patients with non-traumatic injury or did not report OCT data, and in ten the full text was not in English. Twenty-three publications were then excluded after full-text review: in 21 papers, the methods did not specifically define and assess mTBI, one article reported results in paediatric cohort and in one case results had not been published. After title and abstract screening and full-text review, three publications met the criteria for inclusion. The selection process is summarised in Fig. 1.

### Risk of bias

Methodological risk of bias for each study according to the Newcastle-Ottawa Scale [33] is shown in Table 2.

**Table 2:** Main determinants of level of bias within the NEWCASTLE-OTTAWA SCALE Domains for the articles included in this review.

Study	Selection				Comparability	Outcome			Overall
	Representativeness of exposed cases	Selection of the non-exposed cohort	Ascertainment of exposure	Demonstration that outcome of interest was not present at start of study		Assessment of outcome	Was follow-up long enough for outcomes to occur?	Adequacy of follow-up of cohorts	
Chan et al. [36]	Selected group of users (military veterans)	No description of the derivation of the non-exposed cohort	Structured interview*	Yes*	Study controls were not matched	Secure record*	No follow-up	Complete follow-up*	4*
Gilmore et al. [34]	Selected group of users (military veterans)	Draw from the same community as the exposed cohort*	Structured interview*	Yes*	Study controls for age and additional factors (sex, education level, hypertension, diabetes, hazardous drinking, PTSD)*	Secure record*	Yes*	11% lost to follow-up and no description of those lost	6*
Kumar Das et al. [35]	Somewhat representative of the average civilian in the community*	No non-exposed cohort	Structured interview*	Yes*	No control	Secure record*	Yes*	10% lost to follow-up and no description of those lost	5*

*PTSD* posttraumatic stress disorder; *one score.

### Study characteristics

Study characteristics can be found in Table 3.

**Table 3:** Study Characteristics.

Study	Year	Design	Location	Setting	Sample size	Mean age (y) ± SD (range)	Female (%)	Target population	Severity	Diagnostic criteria	Causes of TBI	Time since TBI	OCT type	OCT time points	Comparator
Chan et al. [36]	2019	Retrospective	USA	Single centre	19	37.0 ± 11.8 (27 - 67)	1 (5.3)	Military (veterans)	Mild	VA/DoD	53% blast, 42% blunt, 5% blunt + blast	At least 6 months	Spectralis SD-OCT	Once	4 healthy male controls. Mean age 27± 1years
Gilmore et al. [34]	2020	Prospective longitudinal	USA	Minneapolis VA Healthy Care System	69	50 ± 10.3	7 (10.1)	Military (veterans)	Mild	Mayo	Blast and non-blast	Median (range) 17 (0.5–59) years	Cirrus SD-OCT	Median 4 (3 or more included in analysis)	70 matched healthy controls
Kumar Das et al. [35]	2022	Prospective longitudinal	India	Single Tertiary healthy care hospital	60	N/A (63.4% less than 45 years old)	24 (40)	Civilians	Mild	WHO	Doesn’t specify	7 days	Cirrus HD-OCT	7 days, 3 months, 6 months, 1-year post trauma	None

*VA/DoD* United States Department of Veterans Affairs/Department of Defense, *WHO* World Health Organisation, *SD-OCT* Spectral Domain-Optical Coherence Tomography, *HD-OCT* High Resolution Spectral Domain-Optical Coherence Tomography.

The largest study was by Gilmore et al. [34], assessing 69 mTBI patients (diagnosed according to Mayo diagnostic criteria) and 70 healthy controls, who were well matched for age, sex, education, hypertension, diabetes, hazardous drinking and posttraumatic stress disorder. Kumar Das et al. [35] included 60 mTBI patients (according to WHO criteria), but no control group; and Chan et al. [36] assessed 19 patients (according to VA/DoD criteria) and four healthy male controls. Two studies had a longitudinal design [35, 36], whereas Chan et al. [36] did not report longitudinal data.

Two studies were conducted in the USA [34, 36], and one in India [35]. Two studies included military veterans with chronic mTBI, while one [35] recruited civilians with acute mTBI (i.e. within seven days of incident). The age range for Gilmore [34] and Chan et al. [36] ranged from 27 to 60 years and a minority (5–10%) were female. Kumar Das et al. [35] did not specify the mean age and range for their cohort, only stating that 63.4% were younger than 45 years old. They also had a higher percentage of female participants at 40%. Two of the studies recruited patients from a single centre, with Gilmore et al. [34] recruiting from multiple centres. Gilmore et al. [34] and Chan et al. [36] both recruited blast and non-blast injuries, with Chan et al. [36] being the only study reporting percentages of those with blast injuries. The mechanism of injury was not reported by Kumar Das et al. [35]. There were no ethnicity or patient-reported outcome measures reported in any of the studies [34–36].

### Retinal nerve fibre layer

Heterogeneity in methodology and reporting prevented meta-analysis and we therefore provide a narrative synthesis.

Across all studies 148 patients with mTBI underwent OCT imaging. Of these 136 were included in RNFL analysis. Gilmore et al. only included those who had three or more time points of RNFL data into their final analysis (63 TBI and 61 control group) [34]. Kumar Das et al. lost six to follow-up and two patients were one-eyed, but they did not report if those lost to follow-up were included in final analysis [35]. Chan et al. did not report patients lost to follow-up or excluded from analysis [36].

The cross-sectional data reported by Chan et al. [36] found that the mTBI cohort had an average RNFL of 114 μm and the healthy cohort 120.5 μm. *P* values were reported for each sector, showing a significant difference for the RNFL in the temporal sector, *P* = 0.04, although, overall, there was no significant difference in RNFL across all sectors, *P* = 0.333.

The average RNFL yearly change after mTBI is shown in Table 4. Kumar Das et al. [35] found an average 18 μm decrease in RNFL during the initial 12-month period after mTBI, defining a “reduction in RNFL” as a decrease >20%, and a decrease > 30% as a “significant loss of thickness”, they assessed for RNFL thickening as well. Whilst there were no patients with RNFL thickening at 12 months, they reported 3.7% had RNFL thickening at three months after injury.

**Table 4:** Retinal nerve fibre layer and ganglion cell layer changes.

Study	Year	RNFL			GCL		
		Average μm/y mTBI	Average μm/y control	*p* value	Average μm/y mTBI	Average μm/y control	*p* value
Kumar Das et al. [35]	2022	−18.00	No control	N/A	No GCL	No GCL	N/A
Gilmore et al. [34]	2020	−1.47	−0.31	0.004	−0.17	−0.02	0.16
Chan et al. [36]	2019	Not longitudinal	Not longitudinal	N/A	Not longitudinal	Not longitudinal	N/A

*RNFL* retinal nerve fibre layer, *GCL* ganglion cell layer, *mTBI* mild traumatic brain injury, *N/A* not applicable.

Gilmore et al. followed up patients over a five year period; they showed a significant decrease in average yearly RNFL change in mTBI group (average = −1.47 μm/year), whereas yearly change was −0.31 μm/year in the control group, *P* = 0.004 [34]. They described a yearly RNFL change, with any RNFL change above zero defined as ‘thickening’ and below zero as ‘thinning’. The yearly RNFL slope (μm /year) for the mTBI cohort showed that 81.0% (51/63) had thinning of their RNFL and 19.0% (12/63) showed RNFL thickening. The healthy controls had more participants with RNFL thickening (42.6%, 26/61) and less with RNFL thinning (57.4%, 35/61). However, the baseline RNFL thickness was significantly thicker in the mTBI cohort vs controls (98.4 μm vs 93.8 μm, *P* = 0.01),

### Ganglion cell layer

Of the 148 mTBI patients who underwent OCT imaging, 63 patients had GCL thickness reported. Only Gilmore et al. [34] reported GCL thickness and as mentioned previously, Gilmore et al only included those who had three or more points of RNFL data in their final analysis. GCL changes are shown in Table 4.

Chan et al. [36] did not report GCL averages, but instead sectors. None for the GCL sectors showed any significant differences from the healthy cohort. Kumar Das et al. [35] did not record GCL changes in their cohort. Gilmore et al. [34] showed no significant difference in average yearly GCL change: −0.17 μm/year in the mTBI group and −0.02 μm/year in the control group, *p* = 0.16.

### Other visual measures

All three papers reported visual function measures [34–36]. In Gilmore et al.’s paper, contrast sensitivity displayed statistically significant changes over time in the lowest spatial frequency for monocular vision and at 12 cycles per degree spatial frequency for binocular vision. Visual acuity changes over time did not significantly differ between groups, whereas the changes for visual field mean deviation (yearly negative slope) and pattern standard deviation (yearly positive slope) were statistically significant when both eyes averaged together. A lower visual field mean deviation and lower pattern standard deviation is indicative of visual field loss. Visual field loss was also shown by Kumar Das et al. [35], with 44% of eyes having a visual field index (VFI) of less than 80% at three month post injury (<80% considered a “significant visual field defect”), which was the peak period for developing visual field defects, with stabilisation from six months. The possibility of developing visual field defects (VFI < 80%) became statistically significant when there was >30% decrease in RNFL. The most common visual field defect reported was scatter field defect (27.4% at 3 months, 55.6% no defect). Visual function impairment was significantly worse in those with a recorded VFI < 80% (*P* < 0.001). Forty-three percent of participants had visual symptoms related to visual field loss three months post injury; and by one year this reached 47%. Forty-seven percent of participants in Chan et al. [36] reported mild visual symptoms, including blurred vision, photophobia, floaters and flashes. Additionally, they reported that amongst their cohort there were normal visual fields or ‘mild defects’, no relative afferent pupillary defect, normal colour vision, and mild or no optic disc pallor.

## Discussion

This systematic review on OCT metrics in mTBI found consistent longitudinal RNFL thinning after mTBI, but no significant GCL changes. There was also evidence for clinically meaningful correlations between visual acuity, visual field patterns and RNFL thinning [34, 35].

Perhaps surprisingly, there were no correlations between RNFL and GCL: loss of RNFL should reflect loss of retinal ganglion cell (RGC) axons, with associated degeneration of the RGC cell bodies detected as GCL thinning. This lack of association may be explained if we hypothesise different phases of injury response: initial injury could causes RGC axonal oedema, which would be detected as RNFL thickening (reported by Kumar Das et al. [35] at three month post-injury) compared to pre-injury OCT. Subsequent RGC degeneration could reflect mild traumatic optic neuropathy and be associated with RNFL thinning later after mTBI [34]. Late gliotic changes at the optic nerve head causing RNFL thickening in long-term follow-up may also play a role. The lack of correlation between RNFL and GCL findings could then be explained by the lack of oedema and gliosis affecting the GCL, rendering RNFL a more sensitive marker of visual damage in mTBI.

Other studies utilising OCT have been carried out in patients with moderate and severe TBI. To maintain homogeneity among the participants included in this systematic review, we did not include studies that assessed moderate or severe TBI or in whom injury was defined as “concussion”, but without specified diagnostic criteria. Results from moderate and severe TBI studies are, however, consistent with those highlighted in this review. For instance, additional evidence comes from case studies demonstrating thinning of the RNFL for moderate/severe TBI [37, 38]. When looking at concussion, Leong et al. [39] demonstrated that, in professional collision sport athletes, the average RNFL thickness was a significant predictor of athlete vs control status. Athletes demonstrated a 4.8μm thinning of their RNFL compared to controls*, P* = 0.01, most notably differences were seen in boxers [39, 40].

There have been some experimental animal studies using TBI models and assessing the retina. Animal models are designed to produce a homogenous injury, along with controlling for baseline characteristics. Whilst this does not fully replicate the human population, it allows study of the pathophysiology underlying TBI [41]. Loss of retinal ganglion cells have been detected in blast injury models, with decreased RNFL thickness three months after injury compared to healthy control mice [42], and a 24% decrease in total retinal thickness in a repetitive mTBI mouse model [43], attributed to inner retinal thinning, when compared to controls, *P* < 0.0001.

Only three publications met the inclusion criteria. Of the three papers, two had longitudinal data. We were unable to carry out a meta-analysis due to the small number of included publications and heterogeneity of the reported data. There was heterogeneity in study design (cross-sectional vs longitudinal), differences in assessed populations (civilians vs military) and heterogeneity of injury type. Important heterogeneity between studies was due to discrepancies in the diagnostic criteria used. For instance, Kumar das et al. [35] used the WHO criteria for mTBI diagnosis which does not include brain imaging. Therefore, six patients were revealed to have tiny contusions involving the frontal and cerebellar region (but none involving the optic pathway); these six patients were included in the final analysis. Gilmore et al. [34] used the MAYO TBI Severity Classification System which does include brain imaging, and also differs from the VA/DoD criteria in that it does not include altered level of consciousness. Chan et al. [36] uses the VA/DoD criteria. Another limitation of current studies is under-representation of female population: only 32 patients out of a total of 148 were female, which may relate to recruitment of a young cohort.

Although initial OCT investigations in mTBI are promising, this systematic review highlights that there are still gaps in current literature surrounding OCT changes after mTBI. Many publications had to be excluded as they did not report TBI severity, with cohorts populated with individuals of varying severity or type of brain injury. Therefore, more large prospective longitudinal trials are needed including clear definition of mTBI, detailed description of injury type (when available) and clinical phenotyping of symptoms following TBI. In conclusion, there is consistent evidence that mTBI affects the RNFL, with associated disturbances is visual function [34, 35]. There may therefore be potential in the future to use OCT to measure RNFL as a biomarker for mTBI affecting the visual system.

### Supplementary information

PRISMA
